# Predictors of exclusive breastfeeding in the first six months: four consecutive surveys in a tertiary hospital in Lithuania

**DOI:** 10.1186/s13006-021-00364-6

**Published:** 2021-02-24

**Authors:** Vaidilė Jakaitė, Aurelija Peštenytė, Jolita Zakarevičienė, Vilija Sniečkuvienė, Viktorija Žitkutė, Diana Ramašauskaitė, Gintautas Domža

**Affiliations:** 1 Centre of Obstetrics and Gynecology, Vilnius University Hospital Santaros Klinikos, 2 Santariškių St, LT-08661 Vilnius, Lithuania; 2 Clinic of Obstetrics and Gynecology of the Institute of Clinical Medicine, Faculty of Medicine of Vilnius University, Vilnius, Lithuania; 3 Clinic of Obstetrics and Gynecology, Vilnius City Clinical Hospital, Vilnius, Lithuania

**Keywords:** Exclusive breastfeeding, Breastfeeding on demand, Six months of life

## Abstract

**Background:**

There are little up-to-date data available on the duration of exclusive breastfeeding in Lithuania. The aim of our study was to examine the factors that could influence exclusive breastfeeding during the first 6 months of life.

**Methods:**

In 2016, a survey was conducted at the Obstetrics and Gynecology Clinic of Vilnius University Hospital, Santaros Klinikos. Women in postnatal wards were opportunistically offered questionnaires and later followed up by telephone interviews at 6 weeks, 3 months, and 6 months postpartum. We used binary logistic regression to determine the factors that impacted exclusive breastfeeding during the first 6 months following childbirth.

**Results:**

Of 475 eligible women that were approached, a total of 447 women were recruited, with response rates of 76.1, 71.4 and 67.0% at 6 weeks, 3 months, and 6 months postpartum, respectively. The prevalence of exclusive breastfeeding through the 6 month postpartum period was 39.8%. Exclusive breastfeeding during days 2 to 4 postpartum was positively influenced by factors such as a natural childbirth, the practice of breastfeeding on demand and maternal self-confidence in breastfeeding. Subsequently, exclusive breastfeeding on demand in the immediate postpartum period and exclusive breastfeeding for up to 3 months were associated with successful exclusive breastfeeding up to 6 months. However, the adverse factors that limited the success and duration of exclusive breastfeeding included free samples of human milk substitutes or advertising at primary healthcare centers 6 weeks after childbirth, pacifier use 6 months after childbirth, as well as amniotomy for labor induction.

**Conclusions:**

Our research demonstrated that exclusive breastfeeding is impacted in both directions by a range of factors during particular periods after delivery. One of the novel findings was the adverse influence of amniotomy for labor induction on exclusive breastfeeding rates. Taking into account diverse factors influencing exclusive breastfeeding and the absence of a single way to promote it, there is a crucial need to increase the incidence of exclusive breastfeeding until infants reach the age of 6 months.

**Supplementary Information:**

The online version contains supplementary material available at 10.1186/s13006-021-00364-6.

## Background

Mother’s own milk has historically been deemed the perfect food for babies for the first 6 months. It is not only biologically appropriate for the growth and development of the infant and the competency of their immune system but also beneficial in multiple ways for the breastfeeding woman, her family, and the general public via the health system [1–3].

According to the World Health Organization (WHO) recommendations, infants should be exclusively breastfed for the first 6 months of life, and after starting complementary feeding with the introduction of solid food or liquids other than maternal breast milk, breastfeeding should last up to 2 years or more [4]. Exclusive breastfeeding is the feeding of infants only on breast milk (including expressed milk or milk from a wet nurse) without giving any other liquids or solids, even water, with the exception of medicines, minerals, vitamins or oral rehydration solution. Partial breastfeeding is feeding on breast milk with supplementation with any solid, semisolid foods or liquids, including nonhuman milk and infant formula food [5].

Research shows that a lack of breastfeeding and, in particular, a lack of exclusive breastfeeding leads to an increased risk of morbidity and mortality in infants and children [6]. The short- and long-term benefits of breastfeeding have been proven not only for a healthy start to an infant’s life, but also for maternal health and quality of life. Antibodies produced with colostrum protect against diarrhea and pneumonia, and breastfed infants are less likely to suffer from inflammation of the middle ear or urinary tract infections [7]; all of these may be the cause of sudden infant death [8]. In addition, dental malocclusion in non-breastfed infants is more frequently observed than that in breastfed infants. Higher IQs and larger household incomes have been observed among adults with a breastfeeding history, and they are less likely to suffer from health ailments such as obesity, type 2 diabetes mellitus and other cardiovascular diseases. Breastfeeding women themselves are less likely to develop breast and ovarian cancer, type 2 diabetes, cardiovascular diseases, rheumatoid arthritis, osteoporosis or postnatal depression [3, 6, 9]. After giving birth, the milk ejection reflex while breastfeeding causes faster uterine involution, which inhibits abundant blood loss. In addition, exclusive breastfeeding protects against unplanned early pregnancies [3], although it is important to note that breastfeeding is no longer considered a reliable form of contraception.

According to statistics, no more than 50% of newborns were exclusively breastfed for 3 months in 24 of the 36 countries of the European region, over the period from 2005 through 2010, while only one of the given countries reported that more than 50% of infants were exclusively breastfed for 6 months [10]. However, the global trend of exclusive breastfeeding is growing, with an increase in the incidence of exclusive breastfeeding up to 5 months from 24.9% in 1993 to 35.7% in 2013. In terms of the duration of exclusive breastfeeding in Lithuania, there is a lack of up-to-date statistical data [7].

Given the positive benefits of breastfeeding, this article aims to elucidate the factors impacting exclusive breastfeeding that should be taken into account to increase the incidence of this practice until the infants reach the age of 6 months.

## Methods

### Design

To identify the factors impacting exclusive breastfeeding and thereby increase the incidence of exclusive breastfeeding until the infants reach the age of 6 months, a cohort study with the application of a prospective survey was performed in one of the largest tertiary referral hospitals in the Baltic States.

### Setting

The study was conducted at the Obstetrics and Gynecology Clinic of Vilnius University Clinic Santaros Klinikos. It is a third-level hospital where approximately 35% of women in the region give birth to their babies. Normally, the parturient women are discharged from the obstetrics unit 2 days after delivery.

### Sample

In 2016, the research was carried out at the Obstetrics and Gynecology Clinic of Vilnius University Clinic Santaros Klinikos on days 2–4 postpartum by applying paper-based questionnaires with follow-up via telephone interviews at 6 weeks, 3 months, and 6 months postpartum. The target population was mother-child dyads during the first 6 months postpartum. The survey included 447 mothers who met the inclusion criteria: maternal age of 18–45 years; primiparous or multiparous; childbirth after singleton pregnancy of over 34 weeks’ gestation; and able to give voluntary consent to participate in the survey. The exclusion criteria were as follows: refusal to participate in the study or being unreachable by telephone; multiple pregnancies; childbirth after pregnancy of less than 34 weeks’ gestation; stillbirth or an experience of death of a newborn or an infant.

### Ethical considerations

All women who met the inclusion criteria were approached and offered participation. Those who agreed to participate provided voluntary signed consent and could withdraw at any time.

### Measurements

The women were surveyed using three questionnaires administered by the authors. The questionnaires were compiled on the basis of the scientific literature regarding the factors influencing breastfeeding as well as the Edinburgh Postnatal Depression Scale (EPDS) and Antonovsky’s sense of coherence scale. The questions involved breastfeeding particulars (time to first breastfeeding, feeding method, breastfeeding duration, intervals and times of breastfeeding during the last 24 h, use of breast milk substitutes, etc.), maternal preparation (previous breastfeeding experience, attendance of lectures for pregnant women, reading breastfeeding literature), information on the support available from relatives or medical personnel, and intention to breastfeed (the participants were asked whether and how long they were going to breastfeed their babies at all four surveys). Additional data on the course of pregnancy and childbirth, including antepartum diagnoses and the health status of the neonate and mother, were collected from medical records. In terms of the information on the medications used during labor and the pregnancy, the type and dose of analgesics as well as the use of oxytocin and prostaglandins in labor was recorded, while information on the doses of other medications used during labor and pregnancy was not recorded.

### Data collection

The interviews were carried out four times: first, the respondents were interviewed 2–4 days after childbirth in the hospital, and the data from their childbirth histories were collected; second, a telephone survey was conducted 6 weeks later, followed by repeated telephone surveys at the 3rd and 6th months after childbirth.

The initial interview was performed 2–4 days after childbirth in the postpartum wards or examination rooms within the obstetrics unit. The mothers were familiarized with the aims and methods of the study, and written consent was obtained. The women were questioned in person and completed three questionnaires, and relevant supplementary data were collected from medical records. The questionnaires were as follows: one compiled by us by utilizing the findings from the literature, EPDS, and Antonovsky’s internal coherence validation scales. The follow-up interviews were conducted via telephone. The data entry was checked by the authors entering the data from paper documents into a computer and double-checking the data.

### Data analysis

The data were collected and archived using a standard spreadsheet tool (MS Excel), and the analysis was performed using SPSS 20.0 (Statistical Package for Social Sciences). Descriptive statistics were applied to present the background of participants, and the associations between various factors and exclusive breastfeeding were investigated using logistic regression models. Binary logistic regression was chosen for the analysis, as most of the variables were nominal. A repeated measures method was not used because it is mostly applied to interval variables. Although survival could have been a suitable method to use, the exact time when the respondents ceased to exclusively breastfeed was not known. There were individual models for different times of the study: 2–4 days, 6 weeks, 3 months and 6 months after childbirth. Using the backward (likelihood ratio; LR) method of binary logistic regression, the likelihood ratios of significant factors were determined. The likelihood ratio test was used because it shows how likely the event is and may assist in decision making regarding predictor promotion. The binary outcome variable was exclusive breastfeeding, and the predictor variables and numbers entered into the logistic regression analyses are listed in Additional file 1. Bivariate analyses and comparisons of categorical and continuous data between the groups were explored with chi-square tests and specified with Fisher’s exact tests or small samples. For interval characteristics, Student’s t and Mann-Whitney U tests were deployed. The odds ratios were reported with 95% confidence intervals. The difference in findings was considered significant when *p* < 0.05.

## Results

Of the 475 eligible women approached, a total of 447 parturient women were recruited. Of the eligible and approached women, 5.9% refused to participate due to personal reasons. As stated above, the follow-up interviews were conducted via telephone. On average, 4.5 calls per participant were needed. At 6 weeks, 341 women answered the telephone and agreed to participate in the survey (the response rate was 76.1%), while 3 and 6 months later, 320 women and 300 women were surveyed (71.4 and 67.0% response rates, respectively). The reasons for women leaving the study included refusal to participate due to personal reasons or not being contacted; no one rejoined. The age range of respondents was between 18 and 45 years (mean age: 30.45 ± 5.11). At all surveys, the focus was on breastfeeding. During the 2–4 days postpartum, 423 (94.6%) breastfeeding women were identified, of which 275 (61.4%) women breastfed exclusively. There were 20 (4.5%) mothers with exclusive formula feeding. After 6 weeks, the number of breastfeeding participants was 279 (81.8%), with 207 (60.7%) exclusively breastfeeding and 66 (19.4%) exclusively formula feeding. The third survey 3 months after childbirth revealed 201 (62.8%) participants exclusively breastfeeding out of 235 (73.4%) and 85 (26.6%) exclusively formula feeding. After 6 months postpartum, 185 women (61.7%) continued to breastfeed, while 115 (38.3%) fed exclusively on formula. Out of 300 respondents who participated in all four surveys, 198 (66.0%) women breastfed exclusively 2–4 days after delivery; 138 (46.0%) after 6 weeks; 124 (41.3%) after 3 months; and 117 (39.8%) up to 6 months.

Binary logistic regression methods were deployed to evaluate the factors that could predispose women to exclusive breastfeeding on days 2–4 after childbirth. The findings showed that natural childbirth was a predictor for exclusive breastfeeding. The health status of women and newborns also influenced breastfeeding; lower rates of exclusive breastfeeding were found in those who had suffered from gestational diabetes. There was strong evidence that healthy neonates were breastfed more frequently. Less important but statistically significant factors related to exclusive breastfeeding were breastfeeding on demand but not at fixed hours, self-confidence in breastfeeding, and previous breastfeeding experience of the mothers (see Table 1).

**Table 1:** Factors at 2–4 days after childbirth influencing exclusive breastfeeding on days 2–4 postpartum using the Backward method (*N* = 398)^a^

Factors	B	***p***	OR	95% CI
Previous breastfeeding experience	0.46	0.00***	1.58	1.19, 2.11
Breastfeeding on demand	0.62	0.03	1.86	1.05, 3.29
Self-confidence in breastfeeding	0.87	0.00***	2.40	1.41, 4.06
Pacifier use	−1.19	0.00***	0.30	0.19, 0.50
Gestational diabetes during this pregnancy	−0.90	0.03	0.41	0.18, 0.93
Natural childbirth (vaginal delivery)	0.98	0.00***	2.67	1.50, 4.77
Healthy newborns^b^	1.46	0.00***	4.29	2.30, 8.02

Note: *p* ≤ .05 represents statistical significance

*Abbreviations*: *B* regression coefficient, *OR* Odds ratio (exponent of the estimate), *95% CI* Lower and upper limits of the OR 95% confidence interval

^a^ Only statistically significant variables are included. All variables are listed in Additional file 1 (Supplementary Tables 1-3).

^b^ Healthy newborns – neonates without congenital anomalies or other pathologies requiring admission to special care during the hospital stay

*** *p* ≤ 01

The results showed that women who breastfed on demand on days 2–4 after delivery were more likely to exclusively breastfeed after 6 weeks postpartum. Conversely, amniotomy for labor induction was found to have negatively impacted the uptake of exclusive breastfeeding, and while that was seen to have weaker evidence than other factors, it was nonetheless a significant predictor. Additionally, pacifier use to soothe the baby after hospital discharge was also negatively associated with exclusive breastfeeding 6 weeks postpartum (see Table 2).

**Table 2:** Factors at 2–4 days and 6 weeks after childbirth determining exclusive breastfeeding for up to 6 weeks using the Backward method (*N* = 275)^a^

Times	Factors	B	***p***	OR	95% CI
2–4 days postpartum	Amniotomy for labor induction	−1.792	0.02	0.17	0.04, 0.80
	Breastfeeding on demand	1.402	0.04	2.84	0.97, 8.28
6 weeks postpartum	Pacifier use	−5.049	0.00**	0.01	0.00, 0.02

Note: *p* ≤ .05 represents statistical significance

*Abbreviations*: *B* Regression coefficient, *OR* Odds ratio (exponent of the estimate), *95% CI* Lower and upper limits of the OR 95% confidence interval

^a^ Only statistically significant variables are included. All variables are listed in Additional file 1 (Supplementary Tables 1–3)

** *p* ≤ .01

Furthermore, we investigated the factors that determined exclusive breastfeeding for up to 3 months postpartum. An association was found that when the midwife or doctor explained and showed the women how to breastfeed, the frequency of exclusive breastfeeding 3 months after childbirth was higher. Nonetheless, the sense of confidence and security while breastfeeding also predicted the likelihood of exclusive breastfeeding in the postpartum period. Conversely, higher scores on the EPDS as well as preexisting anxiety or depression disorders were related to lower rates of exclusive breastfeeding at 3 months postpartum. The factor that had the highest influence on exclusive breastfeeding for up to three months postpartum was exclusive breastfeeding six weeks after childbirth. Family input and medical staff support in relation to breastfeeding also positively predicted exclusive breastfeeding up to three months (see Table 3).

**Table 3:** Factors at 2–4 days, 6 weeks and 3 months after childbirth determining exclusive breastfeeding for up to 3 months using the Backward method (*N* = 223)^a^

Stages	Factors	B	***p***	OR	95% CI
2–4 days postpartum	Breastfeeding on demand	2.05	0.00**	7.73	2.02, 29.57
6 weeks postpartum	Free distribution of milk substitutes or advertising in primary healthcare centers	−2.57	0.02	0.12	0.01, 0.89
3 months postpartum	Exclusive breastfeeding	4.00	0.00**	54.43	11.52, 257.11
6 months postpartum	Pacifier use	−2.70	0.00**	0.07	0.02, 0.24

Note: *p* ≤ .05 represents statistical significance

*Abbreviations*: *EPDS* Edinburgh Postnatal Depression Scale, *B* Regression coefficient, *OR* Odds ratio (exponent of the estimate), *95% CI* Lower and upper limits of the OR 95% confidence interval

^a^ Only statistically significant variables are included. All variables are listed in Addional file 1 (Supplementary Tables 1–3)

** *p* ≤ .01

There was strong evidence that women who exclusively breastfed for three months were more likely to maintain that feeding method until six months postpartum. Breastfeeding on demand immediately after childbirth increased the likelihood of exclusive breastfeeding up to six months by almost eight times. Free distribution or advertising of human milk substitutes in the primary healthcare centers six weeks after childbirth and pacifier use six months after delivery were also among factors that reduced the likelihood of exclusive breastfeeding up to six months (see Table 4).

**Table 4:** Factors at 2–4 days, 6 weeks, 3 months and 6 months after childbirth determining exclusive breastfeeding for up to 6 months using the Backward method (*N* = 256)^a^

Times	Factors	B	***p***	OR	95% CI
2–4 days postpartum	Sense of confidence and security while breastfeeding	1.83	0.02	6.20	1.38, 27.79
	EPDS score	−0.20	0.04	0.82	0.68, 0.99
	Midwife/doctor taught how to breastfeed	−1.732	0.00**	0.18	0.05, 1.07
	Pacifier use	−1.239	0.04	0.29	0.09, 0.92
6 weeks postpartum	Exclusive breastfeeding	4.07	0.00**	58.48	4.21, 811.67
3 months postpartum	Family support in relation to breastfeeding	2.51	0.00**	12.30	2.57, 58.92
	Pacifier use	−1.567	0.04	0.21	0.05, 0.98
	Doctor’s support in relation to breastfeeding	1.43	0.02	4.17	1.29, 13.54

Note: *p* ≤ .05 represents statistical significance

*Abbreviations*: *B* Regression coefficient, *OR* Odds ratio (exponent of the estimate), *95% CI* Lower and upper limits of the OR 95% confidence interval

^a^ Only statistically significant variables are included. All variables are listed in Additional file 1 (Supplementary Tables 1–3)

** *p* ≤ .01

Regarding the predictors of exclusive formula feeding, we also carried out an analysis on the factors affecting exclusive formula feeding on days 2–4 after delivery (see Tables 5 and 6). The statistically significant factors positively predicting exclusive formula feeding were as follows: pacifier use, premature birth, intramuscular pethidine for analgesia, cesarean section and longer time after birth until the first breastfeeding. The statistically significant factors negatively predicting exclusive formula feeding included watching electronic programs on breastfeeding at the hospital, sensing that the knowledge on breastfeeding was sufficient and having longer skin-to-skin contact after childbirth. Support in relation to breastfeeding, including support from the family, doctor, midwife and medical staff in general, also negatively predicted exclusive formula feeding. Healthy neonates, in addition to positively predicting exclusive breastfeeding, were negatively associated with exclusive formula feeding.

**Table 5:** Factors (nominal and ordinal variables) at 2–4 days after childbirth influencing exclusive infant formula feeding on days 2–4 postpartum using the Chi-square test and specified with Fisher’s Exact tests^a^

Factors	Exclusive infant formula feeding			
	No	Yes	χ^**2**^	***p***
Watching electronic program on breastfeeding at the hospital	183 (42.9%)	3 (15.0%)	6.1	0.014
Sense that the knowledge on breastfeeding is sufficient			12.14	0.005
No	126 (29.5%)	13 (65.0%)		
Partially	36 (8.4%)	2 (10.0%)		
Yes	265 (62.1%)	5 (25.0%)		
Family support in relation to breastfeeding	384 (89.9%)	13 (65.0%)	11.95	0.001
Doctor’s support in relation to breastfeeding	333 (78.0%)	10 (50.0%)	6	0.014
Midwife’s support in relation to breastfeeding	325 (76.1%)	10 (50.0%)	6.94	0.008
Medical staff’s support in relation to breastfeeding				
Pacifier use	319 (74.7%)	10 (50.0%)	6	0.014
Teat use	138 (32.3%)	11 (55.0%)	4.42	0.035
Delivery	120 (28.1%)	18 (90.0%)	34.3	< 0.001
Premature			12.52	0.005
Term	23 (5.4%)	5 (25.0%)		
Intramuscular pethidine for analgesia	404 (94.6%)	15 (75.0%)		
Type of delivery	14 (3.3%)	4 (20.0%)	13.82	0.006
Cesarean section				
Vaginal birth			12.84	< 0.01
Healthy newborn^b^	89 (20.8%)	11 (55.0%)		
	338 (79.2%)	9 (45.0%)		
	359 (84.1%)	10 (50.0%)	15.40%	0.001

Note: *p* ≤ .05 represents statistical significance

*Abbreviations*: *χ*^*2*^ chi-square test; *p* level/test of significance

^a^ Only statistically significant variables are included

^b^ Healthy newborn – a neonate without congenital anomalies or other pathologies requiring admission to special care during the hospital stay

**Table 6:** Factors (interval variables) at 2–4 days after childbirth influencing exclusive infant formula feeding on days 2–4 postpartum using the Mann-Whitney U test^a^

Factors	Exclusive infant formula feeding		U	***p***
	No	Yes		
Length of skin-to-skin contact (min)	5.0 (0.0–10.0)	0.5 (0.0–5.0)	2067.0	0.048
Time after birth until the first breastfeeding (min)	60.0 (30.0–180.0)	1440.0 (360.0–1860.0)	159.0	0.001

Note: *p* ≤ .05 represents statistical significance

*Abbreviations*: *U* Mann-Whitney U test, *p* Level/test of significance

^a^ Only statistically significant variables are included

## Discussion

### Type of delivery

To ensure the success of breastfeeding, it is appropriate to consider the WHO guidelines for creating a “baby-friendly” environment. One of the milestones in the first minutes of a newborn’s life is the initiation of breastfeeding, which, if possible, should be started in the first hour without interrupting skin-to-skin contact between the mother and the neonate. This claim has been confirmed by studies showing that women who have given birth via a cesarean section are less likely to be exposed to skin-to-skin contact resulting in a delay in the initiation of breastfeeding [11], a decrease in the ability to latch effectively, a decline in lactogenesis II, and subsequently, lower rates of exclusive breastfeeding [12]. Moreover, women who give birth via a cesarean section are administered higher doses of analgesics, which may also predispose them to a lower incidence of exclusive breastfeeding.

### Gestational diabetes

The woman’s state of health during pregnancy is crucial for starting breastfeeding. Our findings coincide with the existing literature, which suggests a negative impact of gestational diabetes on the initiation of breastfeeding in hospitals and then at home [13, 14]. It is important that in cases of gestational diabetes, breastfeeding should be fully promoted, as exclusive breastfeeding has a beneficial effect on both newborn and maternal glucose metabolism, thereby reducing the risk of developing type 2 diabetes in both individuals in the future [7].

### Human milk substitutes

Human milk substitutes are often introduced not only after detection of a newborn’s physiological deviations from a normal growth trajectory but also due to the psychological health of the mother, such as a lack of self-confidence [15, 16]. Breastfeeding women often feel anxious and concerned about the potential lack of milk production, which prompts the premature and arbitrary introduction of infant formula.

### Previous breastfeeding experience

Another important aspect of exclusive breastfeeding is previous breastfeeding experience. Multiparous women with a previous positive experience tended to exclusively breastfeed much more successfully and for longer periods than their primiparous counterparts or those with hindered experiences [17].

### Breastfeeding patterns

Our findings comply with those indicated in the literature: breastfeeding on demand and not at prescribed hours positively influences the breastfeeding process, with more frequent exclusive breastfeeding observed in the first days and in the first 6 months after childbirth. Similarly, due to oxytocin and prolactin reflexes, frequent and long breastfeeding stimulates lactation and ensures the right amount of milk and fat. Therefore, when the baby receives supplementary feeding with other liquids or is breastfed at prescribed hours, the need for sucking is reduced, the physiological needs of the baby are not met, and lactation may decrease or cease over time [18]. This was also confirmed by the results obtained in our study showing that women who successfully exclusively breastfed for up to 3 months continued breastfeeding for up to 6 months after childbirth.

### Breast milk substitutes

The use of breast milk substitutes such as pacifiers or feeding bottles for neonates or infants is associated with poor breastfeeding outcomes, leading to the infant’s dependence on them. Therefore, if it is necessary to feed the infants with expressed milk, it is recommended to feed them with a cup or syringe but not a feeding bottle [19]. Our data showed that pacifier use led to a lower incidence of exclusive breastfeeding and breastfeeding in general [20]. Similarly, when using a pacifier, breastfeeding is terminated earlier than in cases without its use [21]; therefore, not using breast milk substitutes can lead to a positive effect on breastfeeding and its duration [22].

### Amniotomy for labor induction

These results demonstrate a negative association between amniotomy for labor induction and exclusive breastfeeding at 6 weeks postpartum. We consider that induced labor may be more painful, linked to other medical interventions such as labor augmentation with oxytocin or prostaglandins and is more frequently terminated by cesarean section. The negative labor experience decreases self-confidence, instigating negative emotions that may reduce a wish to breastfeed. Other authors have more frequently analyzed negative influences of the whole labor induction on breastfeeding rather than separate methods, which may have resulted in greater errors in calculation [23]. In our investigation, we differentiated particular methods of labor induction. The results did not show any association between the administration of prostaglandins and/or oxytocin and the rate of exclusive breastfeeding but revealed a negative influence of amniotomy in particular.

### Depressive symptoms

In our study, none of the respondents reported prescriptions for any antidepressants or a diagnosis of postnatal depression; however, exclusive breastfeeding was less prevalent among those who demonstrated depressive symptoms with lower EPDS scores. A myriad of other authors has drawn similar conclusions that women showing such symptoms cease exclusive breastfeeding during the first months postpartum more often than women without these symptoms. The same conclusions were also drawn by other authors [16, 24]. Depressive symptoms lead to low self-esteem, the absence of self-confidence, and a lack of belief to be capable of feeding a baby solely on breastmilk, result in more frequent use of breast milk substitutes. Such women tend to be influenced by the environment more easily; therefore, after detecting depressive tendencies and susceptibility to anxiety, it is especially important to support and provide them with information about the benefits and importance of breastfeeding for both mothers and babies.

### Psychological support

Psychological support from relatives and medical staff in relation to breastfeeding not only increases the likelihood of exclusive breastfeeding but also prolongs its duration [18, 24, 25]. Breastfeeding women have a vision that they reach for based on family and healthcare professionals’ expectations. However, as time passes, some women find their own effort and determination insufficient. At that moment, it is of crucial importance to feel the support and encouragement provided by their closest people, especially the spouse, to not give up but continue breastfeeding. Most likely for this reason, the positive influence of support in relation to breastfeeding became apparent only in the third month after childbirth. The stance and opinion of medical staff with whom women communicate after hospital discharge is no less important [26]. With full and positive support, women exclusively breastfeeding longer.

### Marketing of human milk substitutes

Our results coincide with the findings of other authors, that the marketing of breast milk substitutes influences breastfeeding behaviors. In our investigation, women exposed to such advertisements were less likely to breastfeed for the first 6 months after childbirth [27].

### Routinely administered medications

Synthetic prostaglandins for labor induction, oxytocin for labor augmentation, epidural analgesia and intramuscular or intravenous analgesics administered routinely during or after childbirth may play roles in the success of breastfeeding. There is evidence of associations between epidural analgesia, intramuscular opioid analgesia, ergometrine, and oxytocin alone or in combination with ergometrine for the prevention of postpartum hemorrhage and lower breastfeeding rates [28]. However, prospective studies evaluating the risk of medication use on exclusive breastfeeding are scarce. The results of our study did not show any association between the administration of synthetic prostaglandins or oxytocin and exclusive breastfeeding rates or between epidural analgesia or any doses of intramuscular or intravenous analgesics administered during labor and exclusive breastfeeding rates.

### Recommendations

To ensure exclusive breastfeeding during the first 6 months after childbirth, we suggest promoting the salient factors identified in our study and educating the public on the benefits and importance of breastfeeding, encouraging women to breastfeed on demand, avoiding the use of breast milk substitutes for neonates and infants, and encouraging relatives and medical staff to support the mother in breastfeeding. In the case of cesarean section and complicated childbirth, we recommend, whenever possible, ensuring the longest and most continuous skin-to-skin contact between the mother and the neonate.

### Limitations

The limitations of our investigation include the limitations of the methods. The data collection was performed from a single hospital, while the data obtained from participants in several hospitals would have better reflected national tendencies. Another limitation of the study was volunteer bias. Volunteer bias is known as systematic error because of the differences between those who choose to participate in a study and those who do not [29]; in our study, mothers who already had difficulties breastfeeding may have refused to participate. Moreover, there were relatively large numbers of respondents lost to follow-up at the telephone surveys. In particular, the mothers who had already stopped exclusively breastfeeding might have not responded, which may have also affected the results. We also could not reject the possibility of error due to deceitful or inaccurate answers. The individual reasons why the subjects left the study were also not recorded. Finally, although the type and dose of analgesics in labor were recorded, data on the doses of other medications administered during labor and pregnancy were not complete and may have altered the results. For these reasons, the results should be evaluated critically.

## Conclusions

In summary, we have found that natural childbirth (vaginal delivery), absence of gestational diabetes in the current pregnancy, an objectively healthy newborn, breastfeeding on demand, reported maternal self-confidence in breastfeeding, and previous breastfeeding experience are related to exclusive breastfeeding on days 2–4 after childbirth. Additionally, the main statistically significant factor influencing exclusive breastfeeding for up to 6 weeks after childbirth is breastfeeding on demand on days 2–4 after childbirth and, with a primarily negative impact on exclusive breastfeeding is amniotomy for labor induction. The main statistically significant factors influencing exclusive breastfeeding for up to 3 months after childbirth are as follows: sense of confidence and security while breastfeeding on days 2–4 after childbirth as well as exclusive breastfeeding 6 weeks after childbirth and family and doctor support in relation to breastfeeding at 3 months postpartum. Finally, breastfeeding on demand immediately after delivery and continuous exclusive breastfeeding for up to 3 months postpartum, influence breastfeeding up to 6 months after childbirth. On the other hand, the distribution free of charge and advertisement of human milk substitutes in primary healthcare centers 6 weeks after childbirth and pacifier use 6 months after delivery are factors having an adverse effect on exclusive breastfeeding for up to half a year after childbirth. No associations between epidural analgesia, intravenous analgesics or intramuscular analgesics and exclusive breastfeeding rates were found. These findings suggest what should be taken into consideration to increase exclusive breastfeeding rates.

## Supplementary Information

**Additional file 1: Table S1.** Characteristics of participants (the raw data for nominal variables entered into models). **Table S2.** Characteristics of participants (the raw data for ordinal variables entered into models). **Table S3.** Characteristics of participants (the mean values of interval and ratio variables with a normal distribution and median values of variables with a nonnormal distribution).

## Data Availability

The datasets used and analyzed during the current study are available from the corresponding author on reasonable request.
