# Supplementary material for: Predictors of exclusive breastfeeding in the first six months: four consecutive surveys in a tertiary hospital in Lithuania
Source: Int Breastfeed J. 2021 Feb 24;16:22. doi: 10.1186/s13006-021-00364-6 (PMC7903648; doi:10.1186/s13006-021-00364-6)
Supplement: Supplementary file 1 — Additional file 1: Table S1. Characteristics of participants (the raw data for nominal variables entered into models). Table S2. Characteristics of participants (the raw data for ordinal variables entered into models). Table S3. Characteristics of participants (the mean values of interval and ratio variables with a normal distribution and median values of variables with a nonnormal distribution). [file 13006_2021_364_MOESM1_ESM.docx]

**Supplementary Table 1.** Characteristics of participants (the raw data for nominal variables entered into models)

| Factors | Yes | No |
| --- | --- | --- |
| Parameter (categorical data) | N (%) | N (%) |
| 2-4 days postpartum | |  |
| Exclusive breastfeeding | 275 (61.5) | 172 (38.5) |
| Partial breastfeeding | 148 (33.1) | 299 (66.9) |
| Feeding on extracted breast milk | 14 (3.1) | 433 (96.9) |
| Formula feeding | 20 (4.5) | 427 (95.5) |
| Previous problem – mastitis | 21 (4.7) | 426 (95.3) |
| Previous problem – lactostasis | 22 (4.9) | 425 (95.1) |
| Previous problem – cracked nipples | 62 (13.9) | 385 (86.1) |
| Previous breastfeeding problems | 103 (23.0) | 344 (77.0) |
| Nonbreastfeeding | 24 (5.4) | 423 (94.6) |
| Partial breastfeeding: introduction of other liquids | 7 (1.6) | 93 (98.4) |
| Breastfeeding on demand | 339 (75.8) | 108 (24.2) |
| Breastfeeding at prescribed hours | 97 (21.7) | 350 (78.3) |
| Self-confidence while breastfeeding | 308 (68.9) | 139 (31.1) |
| Sense of confidence and security while breastfeeding | 353 (79.0) | 94 (21.0) |
| Skin-to-skin contact immediately after delivery | 279 (62.4) | 168 (37.6) |
| Skin-to skin contact before the first breastfeeding | 15 (3.4) | 432 (96.6) |
| Skin-to-skin contact immediately after delivery distracted and neonate remains naked on mother’s chest/abdomen | 29 (6.5) | 418 (93.5) |
| Skin-to-skin contact immediately after delivery distracted and swaddled neonate remains near mother’s chest | 242 (54.1) | 205 (45.9) |
| Immediately after delivery and before the first breastfeeding neonate is swaddled | 213 (47.7) | 234 (52.3) |
| Midwife/doctor has taught about correct breastfeeding positions | 269 (60.2) | 178 (39.8) |
| Midwife/doctor has taught about correct latching | 263 (58.8) | 184 (41.2) |
| Midwife/doctor has taught about breastfeeding duration and time intervals between breastfeeding | 267 (59.7) | 180 (40.3) |
| Attendance at lectures for pregnant woman during this pregnancy | 139 (31.1) | 308 (68.9) |
| Attendance at lectures for pregnant woman at Vilnius University Hospital Santaros Klinikos during this pregnancy | 76 (17.0) | 371 (83) |
| Attendance at lectures for pregnant woman during previous pregnancy | 83 (18.6) | 364 (81.4) |
| Attendance at lectures for pregnant woman at Vilnius University Hospital Santaros Klinikos during this pregnancy | 18 (4.0) | 429 (96.0) |
| Learning about correct breastfeeding positions during the lectures for pregnant woman | 182 (40.7) | 265 (59.3) |
| Learning about correct latching during the lectures for pregnant woman | 182 (40.7) | 265 (59.3) |
| Learning about breastfeeding duration and time intervals during the lectures for pregnant woman | 176 (39.4) | 271 (60.6) |
| Reading additional literature on breastfeeding | 109 (24.4) | 338 (75.6) |
| Watching electronic program on breastfeeding at the hospital | 186 (41.6) | 261 (58.4) |
| Husband’s support in relation to breastfeeding | 307 (68.7) | 140 (31.3) |
| Mother’s support in relation to breastfeeding | 275 (61.5) | 172 (38.5) |
| Mother’s-in-law support in relation to breastfeeding | 139 (31.1) | 308 (68.9) |
| Sister’s support in relation to breastfeeding | 113 (25.3) | 334 (74.7) |
| Friend’s support in relation to breastfeeding | 168 (37.6) | 279 (62.4) |
| Family support in relation to breastfeeding | 397 (88.8) | 50 (11.2) |
| Currently encountering stress | 152 (34.0) | 295 (66.0) |
| Abuse experience | 14 (3.1) | 433 (96.9) |
| Doctor’s support in relation to breastfeeding | 343 (76.7) | 104 (23.3) |
| Midwife’s support in relation to breastfeeding | 335 (74.9) | 112 (25.1) |
| Medical staff’s support in relation to breastfeeding | 329 (73.6) | 118 (26.4) |
| Advertisement or free distribution of breast-milk substitutes in the hospital | 86 (19.2) | 361 (80.8) |
| Breast substitute distribution in the hospital | 172 (38.5) | 275 (61.5) |
| Pacifier use | 149 (33.3) | 298 (66.7) |
| Bottle feeding | 138 (30.9) | 309 (69.1) |
| Lives with a partner | 376 (84.1) | 71 (15.9) |
| Higher education | 321 (71.8) | 126 (28.2) |
| Currently studying | 42 (9.4) | 405 (90.6) |
| Has a constant job | 385 (86.1) | 62 (13.9) |
| Plans to work full-time | 328 (73.4) | 119 (26.6) |
| Stretch mark appearance | 177 (39.6) | 270 (60.4) |
| Fear of change in looks after delivery | 132 (29.5) | 315 (70.5) |
| Expectations to look better after delivery | 87 (19.5) | 360 (80.5) |
| Plans to breastfeed no less than for 12 months | 157 (35.1) | 290 (64.9) |
| Nonsmoking | 432 (96.6) | 15 (3.6) |
| Lives in a city | 340 (76.1) | 107 (23.9) |
| Preeclampsia | 19 (4.3) | 428 (95.7) |
| Anemia during pregnancy | 108 (24.2) | 309 (75.8) |
| Cholestasis during pregnancy | 5 (1.1) | 442 (98.9) |
| Colpitis | 104 (23.3) | 343 (76.7) |
| Upper respiratory tract infection | 36 (8.1) | 411 (91.9) |
| Taking antibiotics | 35 (7.8) | 412 (92.2) |
| Gestational diabetes | 34 (7.6) | 413 (92.4) |
| Absence of diseases | 307 (68.7) | 140 (31.3) |
| Premature birth | 28 (6.3) | 418 (93.5) |
| Amniotomy for labor induction | 53 (11.9) | 394 (88.1) |
| Synthetic prostaglandins for labor induction | 60 (13.4) | 387 (86.6) |
| No labor induction | 328 (73.4) | 119 (26.6) |
| Oxytocin for labor augmentation | 150 (33.6) | 297 (66.4) |
| Epidural analgesia | 139 (31.1) | 308 (68.9) |
| Intravenous analgesia | 162 (36.2) | 285 (63.8) |
| Cesarean section | 100 (22.4) | 347 (77.6) |
| Vaginal delivery | 347 (77.6) | 100 (22.4) |
| Perineal tearing/episiotomy | 282 (62.6) | 165 (36.9) |
| Vaginal tearing | 4 (0.9) | 443 (99.1) |
| Cervical tearing | 77 (17.2) | 370 (82.8) |
| No tearing | 261 (58.4) | 186 (41.6) |
| Manual removal of placenta | 20 (4.5) | 427 (95.5) |
| Neonate sex female | 197 (44.1) | 250 (55.9) |
| Neonate sex male | 250 (55.9) | 197 (44.1) |
| Healthy neonate* | 369 (82.6) | 78 (17.4) |
| 6 weeks postpartum | | |
| Exclusive breastfeeding | 185 (54.3) | 156 (45.7) |
| Partial breastfeeding | 65 (19.1) | 276 (80.9) |
| Feeding on expressed breast milk | 33 (9.7) | 308 (90.3) |
| Formula feeding | 48 (14.1) | 293 (85.9) |
| Supplementation with liquids other than breast milk | 54 (15.8) | 287 (84.2) |
| Breastfeeding on demand | 219 (64.2) | 122 (35.8) |
| Breastfeeding at prescribed hours | 45 (13.2) | 296 (86.8) |
| Pacifier use | 207 (60.7) | 104 (30.5) |
| Teat use | 113 (33.1) | 228 (66.9) |
| Self-confidence while breastfeeding | 229 (67.2) | 112 (32.8) |
| Sense of confidence and security while breastfeeding | 229 (67.2) | 112 (32.8) |
| Mastitis | 18 (5.3) | 323 (94.7) |
| Lactostasis | 46 (13.5) | 295 (86.5) |
| Flat/inverted or cracked nipples | 106 (31.1) | 235 (68.9) |
| Absence of breastfeeding | 43 (12.6) | 298 (87.4) |
| Family support in relation to breastfeeding | 246 (72.1) | 95 (27.6) |
| Encountering stress | 75 (22.0) | 266 (78.0) |
| Doctor’s support in relation to breastfeeding | 198 (58.1) | 143 (41.9) |
| Free distribution of breast-milk substitutes in primary health care centers (PHCC) | 29 (8.5) | 312 (91.5) |
| Free distribution of breast substitutes in primary health care centers (PHCC) | 17 (5.0) | 324 (95.0) |
| Additional literature on breastfeeding | 148 (43.4) | 193 (56.6) |
| Sense that the knowledge on breastfeeding is sufficient | 243 (71.3) | 98 (28.7) |
| Smoking | 7 (2.1) | 334 (97.9) |
| Plans to return to work | 21 (6.2) | 320 (93.8) |
| Currently works | 26 (7.6) | 315 (92.4) |
| 3 months postpartum | | |
| Exclusive breastfeeding | 192 (60.0) | 128 (40.0) |
| Partial breastfeeding | 36 (11.3) | 284 (88.7) |
| Feeding on expressed breast milk | 33 (10.3) | 287 (89.7) |
| Formula feeding | 97 (30.3) | 223 (69.7) |
| Feeding on puree/porridge | 4 (1.3) | 316 (98.8) |
| Feeding on cow milk | 1 (0.3) | 319 (99.7) |
| Supplementation with liquids other than breast milk | 117 (36.6) | 203 (63.4) |
| Breastfeeding on demand | 203 (63.4) | 117 (36.6) |
| Breastfeeding at prescribed hours | 40 (12.5) | 280 (87.5) |
| Pacifier use | 199 (62.2) | 121 (37.8) |
| Teat use | 134 (41.9) | 186 (58.1) |
| Self-confidence while breastfeeding | 118 (36.9) | 202 (63.1) |
| Sense of confidence and security while breastfeeding | 117 (36.6) | 203 (63.4) |
| Mastitis | 14 (4.4) | 306 (95.6) |
| Lactostasis | 31 (9.7) | 289 (90.3) |
| Flat/inverted or cracked nipples | 27 (8.4) | 293 (91.6) |
| Family support in relation to breastfeeding | 139 (43.4) | 181 (56.6) |
| Encountering stress | 77 (24.1) | 243 (75.9) |
| Doctor’s support in relation to breastfeeding | 204 (63.8) | 116 (36.3) |
| Free distribution of breast-milk substitutes in primary health care centers (PHCC) | 35 (10.9) | 285 (89.1) |
| Free distribution of breast substitutes in primary health care centers (PHCC) | 29 (9.1) | 291 (90.9) |
| Additional literature on breastfeeding | 135 (42.2) | 185 (57.8) |
| Sense that the knowledge on breastfeeding is sufficient | 251 (78.4) | 69 (21.6) |
| Smoking | 7 (2.2) | 313 (97.8) |
| Plans to return to work | 24 (7.5) | 296 (92.5) |
| Currently works | 35 (10.9) | 285 (89.1) |
| 6 months postpartum | | |
| Supplementation with liquids other that mother’s milk | 197 (65.7) | 103 (34.3) |
| Breastfeeding on demand | 171 (57.0) | 129 (43.0) |
| Breastfeeding at prescribed hours | 25 (8.3) | 275 (91.7) |
| Pacifier use | 205 (64.1) | 95 (31.7) |
| Teat use | 144 (48.0) | 156 (52) |
| Self-confidence while breastfeeding | 194 (64.7) | 106 (35.3) |
| Sense of confidence and security while breastfeeding | 195 (65.0) | 105 (35.0) |
| Mastitis | 10 (3.3) | 290 (96.7) |
| Lactostasis | 27 (9.0) | 273 (91) |
| Flat/inverted or cracked nipples | 15 (5.0) | 285 (95) |
| Family support in relation to breastfeeding | 211 (70.3) | 89 (29.7) |
| Encountering stress | 48 (16.0) | 252 (84.0) |
| Doctor’s support in relation to breastfeeding | 189 (60.0) | 111 (40.0) |
| Free distribution of breast-milk substitutes in primary health care centers (PHCC) | 38 (12.7) | 262 (87.3) |
| Free distribution of breast substitutes in primary health care centers (PHCC) | 32 (10.7) | 268 (89.3) |
| Additional literature on breastfeeding | 115 (38.3) | 185 (61.7) |
| Sense that the knowledge on breastfeeding is sufficient | 148 (49.3) | 152 (50.7) |
| Smoking | 9 (3.0) | 291 (97.0) |
| Plans to return to work | 19 (6.3) | 281 (93.7) |
| Currently works | 28 (9.3) | 272 (90.7) |

* Healthy neonate – a newborn without congenital anomalies or other pathologies requiring admission to special care during the hospital stay.

**Supplementary Table 2.** Characteristics of participants (the raw data for ordinal variables entered into models)

| Parameter (categorical data) | N (%) |
| --- | --- |
| **2-4 days postpartum** | |
| Previous breastfeeding experience |  |
| None | 236 (52.8) |
| Little | 99 (22.1) |
| Medium Significant | 89 (19.9) 23 (5.1) |
| The electronic program on breastfeeding has been |  |
| Not beneficial | 5 (1.1) |
| Partially beneficial | 146 (32.7) |
| Beneficial | 35 (7.8) |
| Sense that the knowledge on breastfeeding is sufficient |  |
| No | 139 (31.1) |
| Partially | 38 (8.5) |
| Yes | 270 (60.4) |
| Expectations for looks |  |
| Worse | 166 (37.1) |
| Better | 80 (17.9) |
| Other | 201 (45.0) |
| Satisfaction in looks |  |
| No | 135 (30.2) |
| Partially | 32 (7.2) |
| Yes | 279 (62.4) |
| Smoking |  |
| No | 402 (89.9) |
| Before pregnancy | 30 (6.7) |
| During pregnancy | 15 (3.4) |
| Intravenous fentanyl for analgesia |  |
| None | 298 (66.7) |
| Once | 123 (27.5) |
| Twice | 24 (5.4) |
| Three times | 2 (0.4) |
| Intramuscular pethidine for analgesia |  |
| None | 429 (96.0) |
| Once | 18 (4.0) |
| Edinburgh Postnatal Depression Scale (EPDS) score |  |
| 0-4 | 218 (48.8) |
| 5-11 | 210 (47.0) |
| >11 | 18 (4.0) |
| Antonovsky’s validation of sense of coherence scale score |  |
| 25-55 | 164 (36.7) |
| 56-65 | 283 (63.3) |
| **6 weeks postpartum** | |
| Satisfaction in looks |  |
| No | 79 (23.2) |
| Partially | 38 (11.1) |
| Yes | 175 (51.3) |
| Edinburgh Postnatal Depression Scale (EPDS) score |  |
| 0-4 | 188 (55.1) |
| 5-11 | 91 (26.7) |
| >11 | 16 (4.7) |
| Antonovsky’s validation of sense of coherence scale score |  |
| 25-55 | 68 (19.9) |
| 56-65 | 226 (66.3) |
| **3 months postpartum** | |
| Satisfaction in looks |  |
| No | 57 (17.8) |
| Partially | 32 (0.1) |
| Yes | 203 (63.4) |
| Edinburgh Postnatal Depression Scale (EPDS) score |  |
| 0-4 | 209 (65.3) |
| 5-11 | 87 (27.2) |
| >11 | 3 (0.9) |
| Antonovsky’s validation of sense of coherence scale score |  |
| 25-55 | 37 (11.6) |
| 56-65 | 262 (81.9) |
| **6 months postpartum** | |
| Satisfaction in looks |  |
| No | 30 (10.0) |
| Partially | 39 (13.0) 231 (77.0) |
| Yes |  |
| Edinburgh Postnatal Depression Scale (EPDS) score | 217 |
| 0-4 | -72.3 |
| 5-11 | 80 (26.7) |
| >11 | 3 (1.0) |
| Antonovsky’s validation of sense of coherence scale score |  |
| 25-55 | 29 (9.7) |
| 56-65 | 270 (90.0) |

**Supplementary Table 3.** Characteristics of participants (the mean values of interval and ratio variables with a normal distribution and median values of variables with a nonnormal distribution)

| Parameter (continuous data) | Mean (SD)/Median (25^th^, 75^th^ centiles) | Full range |
| --- | --- | --- |
| 2-4 days postpartum | | |
| Age | 30.4 (5.1) | 18.0-45.0 |
| Breastfeeding times per day* | 8.0 (6.0, 10.0) | 0.0-27.0 |
| Length of time between breastfeeding (hours)* | 2.5 (2.0, 3.0) | 0.0-3.0 |
| Longest time between breastfeeding (hours)* | 4.0 (3.0, 5.0) | 2.0-15.0 |
| Duration of breastfeeding (min)* | 30.0 (20.0, 30.0) | 0.0-120.0 |
| Length of skin-to-skin contact (min)* | 5.0 (0.0, 15.0) | 0.0-120.0 |
| Time after birth until the first breastfeeding (min)* | 60.0 (30.0, 360.0) | 0.0-4320.0 |
| Height (cm) | 168.2 (6.0) | 149.0-187.0 |
| Weight (kg)* | 62.0 (55.0, 74.0) | 35.0-131.0 |
| BMI (weight/height^2^)* | 22.0 (19.8, 25.6) | 15.2-49.3 |
| Weight gain during pregnancy (kg) | 14.6 (5.0) | 1.0-30.0 |
| Number of pregnancies* | 2.0 (1.0, 3.0) | 1.0-9.0 |
| Number of births* | 2.0 (1.0, 2.0) | 1.0-6.0 |
| Number of miscarriages* | 0.0 (0.0, 0.0) | 0.0-4.0 |
| Number of preterm births* | 0.0 (0.0, 0.0) | 0.0-1.0 |
| Pregnancy week* | 39.0 (38.0, 40.0) | 31.0-42.0 |
| Cervical dilation during epidural administration (cm) | 5.2 (1.4) | 1.0-9.0 |
| Pethidine for analgesia (mg)* | 0.0 (0.1, 0.1) | 0.0-200.0 |
| Bupivacaine for analgesia (mg)* | 0.0 (50.0, 75.0) | 0.0-195.0 |
| Lidocaine for analgesia (mg)* | 0.0 (40.0, 70.0) | 0.0-300.0 |
| Length of epidural analgesia (hours)* | 4.0 (2.8, 5.3) | 0.0-8.5 |
| Fentanyl injection times* | 0.0 (0.0, 1.0) | 0.0-3.0 |
| Apgar score at 1 min after birth* | 9.0 (9.0, 9.0) | 6.0-10.0 |
| Apgar score at 5 min after birth* | 10.0 (9.0, 10.0) | 8.0-10.0 |
| Birth height (cm)* | 52.0 (51.0, 54.0) | 42.0-59.0 |
| Birth weight (kg) | 3507.3 (538.7) | 1980.0-5210.0 |
| Edinburgh Postnatal Depression Scale (EPDS) score | 5.1 (3.4) | 0.0-19.0 |
| Antonovsky’s validation of sense of coherence scale score | 56.5 (5.4) | 20.0-65.0 |
| 6 weeks postpartum | | |
| Weight after hospital discharge* | 69.0 (62.8, 78.0) | 46.0-139.0 |
| Weight after 6 weeks postpartum* | 65.0 (59.0, 73.0) | 43.0-148.0 |
| Neonatal weight after hospital discharge | 3319.3 (503.0) | 2100.0-4800.0 |
| Lowest neonatal weight (kg) | 3271.3 (482.9) | 1950.0-4760.0 |
| Time of return to work (months)* | 0.5 (0.0, 1.0) | 0.0-1.5 |
| Work hours per day* | 2.0 (1.0, 4.0) | 0.0-9.0 |
| Edinburgh Postnatal Depression Scale (EPDS) score* | 3.0 (1.0, 6.0) | 0.0-21.0 |
| Antonovsky’s validation of sense of coherence scale score* | 59.0 (56.0, 62.0) | 38.0-65.0 |
| 3 months postpartum | | |
| Weight after 3 months postpartum (kg)* | 65.0 (58.0, 72.0) | 43.0-140.0 |
| Time of return to work* | 1.3 (0.8, 2.8) | 0.0-1.5 |
| Work hours per day* | 2.0 (2.0, 4.4) | 0.0-9.0 |
| Edinburgh Postnatal Depression Scale (EPDS) score* | 3.0 (1.0, 5.0) | 0.0-19.0 |
| Antonovsky’s validation of sense of coherence scale score* | 59.0 (56.0, 62.0) | 38.0-65.0 |
| 6 months postpartum | | |
| Introduction of complementary feeding (month)* | 5.5 (3.0, 6.0) | 0.0-6.0 |
| Weight after 6 months postpartum (kg)* | 62.0 (56.0, 70.0) | 47.0-130.0 |
| Time of return to work* | 1.3 (0.6, 3.0) | 0.0-6.0 |
| Work hours per day | 4.5 (2.2) | 0.0-10.0 |
| Edinburgh Postnatal Depression Scale (EPDS) score* | 3.0 (1.0, 5.0) | 0.0-17.0 |
| Antonovsky’s validation of sense of coherence scale score* | 62.0 (59.0, 64.0) | 49.0-68.0 |

*Nonnormal distribution of the variables.
